# Transcriptomic Analysis Reveals Endometrial Dynamics in Normoweight and Overweight/Obese Polycystic Ovary Syndrome Women

**DOI:** 10.3389/fgene.2022.874487

**Published:** 2022-05-13

**Authors:** Su Liu, Ling Hong, Ruochun Lian, Shan Xiao, Yuye Li, Lianghui Diao, Yong Zeng

**Affiliations:** Shenzhen Key Laboratory of Reproductive Immunology for Peri-implantation, Shenzhen Zhongshan Institute for Reproduction and Genetics, Fertility Center, Shenzhen Zhongshan Urology Hospital, Shenzhen, China

**Keywords:** body mass index, endometrium, inflammatory response, polycystic ovary syndrome, transcriptome

## Abstract

The aim of this work was to identify the transcriptomic characteristics of the endometrium in normoweight and overweight/obese polycystic ovary syndrome (PCOS) potentially underlying the pathogenesis. This study included 38 patients undergoing *in vitro* fertilization: 22 women with PCOS and 16 matched controls. Each of the groups was subdivided into normoweight (body mass index (BMI) < 25 kg/m^2^) and overweight/obese (BMI ≥25 kg/m^2^) subgroups. Endometrium samples were collected in the secretory phase from controls or in a modeled secretory phase using daily administration of progesterone from women with PCOS before *in vitro* fertilization treatment. Transcriptome profiles were assessed by high-throughput RNA sequencing to investigate distinct endometrial gene expression patterns in PCOS. Bioinformatics analyses revealed that the endometrium from PCOS expresses significantly different transcripts encoding endometrial receptivity, inflammatory response, angiogenesis, and energy metabolism. Additionally, our study demonstrated that the differentially expressed genes between normoweight and overweight/obese PCOS are involved in fatty acid metabolism, endometrial decidualization, and immune response. For the first time, we have described the transcriptome characteristics of normoweight and overweight/obese PCOS endometria. Our results indicate different endometrial gene expressions between different subtypes of PCOS and non-PCOS women, which might affect endometrial functions in PCOS patients.

## Introduction

Polycystic ovary syndrome (PCOS) is one of the most common endocrine disorders, affecting approximately 5–15% of women worldwide (Lizneva et al., 2016). The diagnosis of PCOS is mainly based on two of the three main features: oligo- or anovulation, hyperandrogenism (clinical or biochemical), and polycystic ovarian morphology (PCOM) on ultrasound, after exclusion of other causes (Amsterdam ESHRE/ASRM-Sponsored 3rd PCOS Consensus Workshop Group, 2012; Legro et al., 2013). The phenotype of PCOS and its impact on reproductive outcomes are profoundly affected by obesity (Salehi et al., 2004).

Ovarian dysfunction is an apparent cause of infertility in PCOS patients (Savaris et al., 2011), which can be treated with ovulation-inducing agents. However, even after ovulation is pharmacologically restored, anovulatory PCOS patients still have reduced cumulative pregnancy rates and exhibit a higher rate of spontaneous miscarriage (Bellver et al., 2011). Therefore, in addition to anovulation, the endometrial environment may also be associated with low fertility in PCOS women. In human reproduction, a receptive endometrium, a functional embryo, and a synchronized dialog between maternal and embryonic tissues are critical for a successful pregnancy (Diedrich et al., 2007; Evans et al., 2016). Previous studies have revealed that several clinical and biochemical factors related to the syndrome can exert a deleterious effect on the endometrium of PCOS women (Giudice, 2006; Shang et al., 2012). Clinical features/characteristics of PCOS may contribute to dysregulation of sex hormone receptor expression and function in the endometrium, increase endometrial insulin resistance, and result in immune dysfunction and altered vascular formations (Palomba et al., 2021; Zhao et al., 2021). Endometrial dysfunction was found to be involved in the increased risk of pregnancy complications (Palomba et al., 2015) and endometrial cancer (Barry et al., 2014) in women with PCOS. Obesity plays an important role in the pathogenesis of PCOS, and approximately 61% of women with PCOS are reported to be overweight or obese (Lim et al., 2012). Studies have suggested that the implantation rate, clinical pregnancy rate, and live birth rate are significantly decreased as body mass index (BMI) increased, despite the embryo quality, suggesting that the endometrium may be the main contributor of the impaired reproductive outcome of these patients (Bellver et al., 2010). Obese PCOS patients showed abnormal levels of phosphatidylcholine, free fatty acids, and polyunsaturated fatty acid metabolites (Li et al., 2017). A previous study also demonstrated that obesity might have a negative effect on endometrial GLUT4 expression in PCOS, indicating that obesity may impair endometrial glucose metabolism (Mioni et al., 2004). Moreover, adiponectin, which is an obesity marker and elicits an insulin sensitizer action, was found to diminish the endometrial level in obese PCOS women (Orostica et al., 2016). Although there have been evidence that obese PCOS patients show endometrial metabolic derangements, many previous studies failed to obtain BMI-matched controls, making it impossible to interpret whether the differences are due to obesity or PCOS.

RNA sequencing analysis is a suitable modality to examine changes in global gene expression in the endometrium in PCOS and may aid in the identification of novel candidate genes that contribute to the etiology of PCOS. To the best of our knowledge, gene expression profiles in the endometrium from normoweight and overweight/obese PCOS women undergoing *in vitro* fertilization (IVF) have not been reported. In this study, the patients, divided into PCOS and control groups, were further subdivided into four subgroups: normoweight PCOS, overweight/obese PCOS, normoweight controls, and overweight/obese controls. Our aim was to determine whether the human mid-secretory phase endometrial transcriptome is altered between PCOS patients and controls, and between the normoweight PCOS and overweight/obese PCOS subgroups at a molecular level during the peri-implantation period. For this purpose, we analyzed the global changes in endometrial gene expression profiles and alterations in genetic networks, signal transduction, metabolic pathways associated with PCOS, and the presence of obesity that may impact endometrial function.

## Materials and Methods

### Patient Recruitment

This study was initially designed to investigate the endometrial dysfunction of infertile women since an inadequate endometrium has been considered a main fertility-determining factor (Strowitzki et al., 2006). Our previous data revealed that women with PCOS had altered endometrial immune cells (Liu et al., 2021). During the study period, we performed the study to investigate the transcriptomic profile of the endometrium in women with PCOS since there were very few studies on the endometrial transcriptomic profile of different subtypes of these patients. This research was designed as a retrospective case–control study. Normal weight was defined as a BMI ≥18.5 kg/m^2^ to <25 kg/m^2^, and overweight and obese were defined as a BMI ≥25 kg/m^2^ according to Asia-specific BMI criteria, which were determined by the World Health Organization Western Pacific Region (WHO Expert Consultation, 2004). Although the metabolic profiles of overweight and obese groups are different, we tend to combine these two groups together in this study as there were very few obese subjects recruited. We identified 22 women with PCOS and 16 regularly menstruating control women who visited Shenzhen Zhongshan Urology Hospital from January 2016 to January 2020. Among these women, 24 were included for transcriptome sequencing as screening: 12 PCOS patients, six normoweight (N-PCOS) with a BMI <25 kg/m^2^ and six overweight/obese (O-PCOS) with a BMI ≥25 kg/m^2^; 12 non-PCOS controls, six normoweight (N-CON) with a BMI <25 kg/m^2^ and 6 overweight/obese (O-CON) with BMI ≥25 kg/m^2^. The other 14 women were included for validation: seven N-PCOS, three O-PCOS, and four N-CON. BMI was measured on their first visit to our center prior to the IVF treatment.

According to the revised 2003 Rotterdam consensus diagnostic criteria for PCOS, the diagnosis of PCOS was based on the association of at least two of the following criteria: 1) oligo-ovulation or anovulation, 2) clinical or biochemical sign of hyperandrogenism, and 3) at least one ovary with polycystic morphology. Patients with other potential causes of anovulation or hyperandrogenemia were excluded. No PCOS patients had evidence of congenital adrenal hyperplasia, Cushing’s syndrome, androgen-secreting tumors, non-classic adrenal hyperplasia, thyroid dysfunction, hyperprolactinemia, type 2 diabetes mellitus, or cardiovascular disease.

The control participants were selected from women who had a live-birth baby after their first IVF or intracytoplasmic sperm injection (ICSI), and the sole cause of marital infertility was male azoospermia based on clinical evaluation. All controls had regular menstrual cycles and normal androgen levels, and none had polycystic ovaries on ultrasound. Patients with endometriosis, endometritis, autoimmune- or thyroid-related disease, abnormal karyotypes, positive infectious disease tests, uterine malformation, and ultrasonographic evidence of hydrosalpinx were excluded from the control group. No subjects had received hormonal treatment or insulin-lowering agents in the previous quarter. This study was approved by the Institutional Review Board of Reproductive Research Ethics Committees of Shenzhen Zhongshan Urology Hospital (Approval number: SZZSECHU-2020023). All the patients were aware of the research objective and signed an informed consent form.

### Laboratory Tests

For each participant, peripheral blood was collected in the morning after an overnight (8–10 h) fast, preferably on cycle day 3 of a natural menstrual cycle in regularly menstruating women or during withdrawal bleeding in amenorrheic women. Basal serum levels for luteinizing hormone (LH), follicle-stimulating hormone (FSH), thyroid-stimulating hormone (TSH), total testosterone (T), and anti-Müllerian hormone (AMH) were determined on menstrual cycle day 2 or 3 in control and PCOS patients by chemiluminescence under a Cobas e601 analyzer (Roche Diagnostics, Germany) using commercial kits, whereas plasma glucose and other biochemical parameters were assayed on a Cobas c501 autoanalyzer (Roche Diagnostics, Germany). For all measurements, the inter- and intra-assay coefficients of variation were within the limits permitted by the manufacturers.

### Endometrial Sample Collection

Hormone replacement therapy cycles were used for enrolled anovulatory PCOS patients, during which estrogen and progesterone were administered consecutively to mimic the endocrine conditions of the endometrium in the nature cycle. The secretory endometrium was obtained after daily progesterone injections for 6–8 days in anovulatory women with PCOS, based on published methods that mimic mid-secretory endometrium (Usadi et al., 2008). Endometrial tissue samples from controls were collected during the mid-secretory phase of the menstrual cycle between day 7 and day 9 after the LH surge. Endometrial biopsies were obtained by soft curettage using an endometrial curette and processed within 2 h after collection. The endometrial samples were divided into two pieces: one was subjected to routine paraffin embedding and the other was snap-frozen in liquid nitrogen and stored in a deep freezer until RNA extraction. Paraffin-embedded endometrial tissues were sectioned at 4 μm thickness. For each sample, one section was randomly chosen for H&E staining to confirm the histologic phase (Supplementary Figure S1).

### Total RNA Extraction and Expression Calculation

For the first batch of 24 recruited samples (including 12 PCOS and 12 controls), total RNA was extracted from the endometrium using TRIzol following the manufacturer’s protocols (Invitrogen, CA, United States). RNA degradation and contamination were monitored on 1% agarose gels. RNA purity was checked using a NanoPhotometer^®^ spectrophotometer (Implen, CA, United States). RNA concentration was measured using a Qubit^®^ RNA Assay Kit in a Qubit^®^2.0 Fluorometer (Life Technologies, CA, United States). RNA integrity was assessed using an RNA Nano 6000 Assay Kit of the Bioanalyzer 2100 system (Agilent Technologies, CA, United States). FeatureCounts v1.5.0-p3 was used to count the read numbers mapped to each gene. FPKM, the expected number of fragments per kilobase of transcript sequence per millions base pairs sequenced, which is currently the most commonly used method for estimating gene expression levels, was calculated based on the length of the gene and read count mapped to this gene. Differential expression analysis of two conditions/groups (two biological replicates per condition) was performed using the DESeq2 R package (1.16.1). DESeq2 provides statistical routines for determining differential expression in digital gene expression data using a model based on the negative binomial distribution. All the differential expression analysis of two conditions was performed using the edgeR R package (3.18.1). The differential expressed genes (DEGs) were determined by two criteria: 1) the fold change between the means of groups was greater than 2.0, and 2) the *p*-value calculated from pooled *t*-test was less than 0.05. The sequencing data were deposited in the NCBI Database (BioProject accessions: PRJNA777962).

### Weighted Gene Co-Expression Network Analysis (WGCNA)

Gene co-expression network analysis was specifically performed on endometrium tissues using the R package WGCNA (V1.68) (Langfelder and Horvath, 2008). The expression matrix was restricted to only expressed genes, when the number of reads sequenced was greater than 10 in a minimum of 24 samples, normalized for samples depth (count per million reads, CPM), and log-transformed, including a pseudo-count of 4 (log_10_ (CPM +4)). Then, the optimal soft threshold for adjacency computation was graphically determined, and we plotted module detection via dynamic tree cutting. For the demonstration of the relationship between different modules, results were visualized using module and eigengene relation heatmap, and the gene co-expression network was extracted and further processed using MCODE in Cytoscape software (V3.6.2) (Shannon et al., 2003). For a distinct and precise representation of the data, genes contained in the significantly relevant modules were extracted and analyzed using Metascape (Zhou et al., 2019).

### GO and KEGG Enrichment Analysis of DEGs

Gene Ontology (GO) enrichment analysis of differentially expressed genes was implemented by the clusterProfiler R package, in which gene length bias was corrected. GO terms with a corrected *p*-value of less than 0.05 were considered significantly enriched by differentially expressed genes.

KEGG is a database resource for understanding high-level functions and utilities of the biological system, such as cell, organism, and ecosystem, from molecular-level information, especially large-scale molecular datasets generated by genome sequencing and other high-throughput experimental technologies (http://www.genome.jp/kegg/). We used clusterProfiler R package to test the statistical enrichment of differential expression genes in KEGG pathways.

### Quantitative Real-Time PCR (qRT-PCR) Analysis

In addition to the 24 sequenced samples, extra 14 independent samples (including 10 PCOS and four controls) were added to measure relative gene expression using qRT-PCR for validation. A measure of 500 ng of endometrium total RNA was used for cDNA synthesis, which was performed using an M-MLV reverse transcriptase kit (Toyobo, Japan) in accordance with the manufacturer’s instructions. Synthesized cDNA was utilized for PCR with primers at optimized cycles (Table S1). qRT-PCR was performed using an ABI QuantStudio™ 5 Real-Time PCR System (Applied Biosystems, CA, United States) with the QuantiTect SYBR Green PCR kit (Toyobo, Hilden, Japan). For comparison of transcript levels between samples, a standard curve of cycle thresholds for several serial dilutions of a cDNA sample was established and then used to calculate the relative abundance of each gene. Values were then normalized to the relative amounts of β-actin. All experiments were performed at least in triplicate for each gene.

### Statistical Analysis

Statistical analyses were performed with Statistical Program for Social Science 25.0 software (SPSS Inc., Chicago, IL, United States). The Kolmogorov–Smirnov test was used to confirm the normal distribution of data. Data were expressed as median with an interquartile range for abnormally distributed data. Mann–Whitney *U* tests were used for evaluating the statistical significance. *p* < 0.05 was considered statistically significant.

## Results

### Clinical Characteristics

The clinical and biochemical characteristics of participants for transcriptome sequencing are listed in Table 1. There was no significant difference between N-PCOS and N-CON groups with regard to age, BMI, basal FSH, total T, TSH, and AMH. They were also similar between O-PCOS and O-CON groups. As expected, in both PCOS and control groups, BMI was significantly higher in overweight/obese women than in normoweight women. In addition, BMI was slightly higher in overweight/obese PCOS than in overweight/obese controls (27.3 vs 26.6 kg/m2, *p* = 0.236) as overweight or obese was more frequent in PCOS women than their non-PCOS counterparts. PCOS patients were characterized by higher LH than BMI-matched controls (in normoweight women: 11.3 vs 3.0 IU/L, *p* = 0.005; in overweight/obese women: 4.7 vs 2.7 IU/L, *p* = 0.023). When we compared the LH/FSH ratio, we found that it was higher among the N-PCOS group than among the N-CON group (1.98 vs 0.85), but this difference was not statistically significant (*p* = 0.103). Fasting glucose and fasting insulin were only available in PCOS women, and there was no significant difference between the N-PCOS and O-PCOS groups.

**TABLE 1:** Characteristics of control women and patients with PCOS.

Characteristic	N-PCOS (*n* = 6)	O-PCOS (*n* = 6)	N-CON (*n* = 6)	O-CON (*n* = 6)	*p*-value^a^	*p*-value^b^	*p*-value^c^	*p*-value^d^
Age (y)	30.0 (25.0–36.0)	29.7 (28.0–30.0)	30.5 (28.0–33.0)	30.7 (24.0–36.0)	0.807	0.695	0.860	0.950
BMI (kg/m2)	21.3 (19.5–23.9)	27.3 (26.4–29.6)	21.0 (18.9–24.2)	26.6 (25.7–27.7)	0.756	0.236	0.000	0.000
FSH (IU/L)	6.9 (3.6–10.8)	5.7 (4.3–8.9)	4.0 (2.0–7.1)	4.1 (2.1–7.2)	0.057	0.204	0.380	0.945
LH (IU/L)	11.3 (3.2–18.8)	4.7 (2.4–6.3)	3.0 (1.7–4.7)	2.7 (1.3–4.3)	0.005	0.023	0.018	0.681
LH/FSH ratio	1.98 (0.43–4.52)	0.85 (0.53–1.30)	0.85 (0.35–1.35)	0.70 (0.50–1.02)	0.103	0.314	0.103	0.354
Total T (ng/ml)	0.5 (0.3–0.8)	0.5 (0.1–1.1)	0.3 (0.1–0.7)	0.2 (0.1–0.4)	0.087	0.237	0.586	0.317
TSH (μIU/ml)	2.2 (1.3–3.2)	2.5 (1.2–3.9)	1.8 (1.5–2.3)	2.6 (1.3–3.7)	0.250	0.916	0.608	0.148
Fasting glucose (mmol/L)	5.3 (5.0–6.1)	5.5 (5.1–6.2)	—	—	—	—	0.555	—
Fasting insulin (μU/mL)	10.7 (5.0–18.0)	13.5 (9.6–21.9)	—	—	—	—	0.285	—
AMH (ng/ml)	3.6 (2.6–5.9)	5.1 (3.4–9.5)	4.0 (1.5–6.6)	3.6 (2.6–5.9)	0.385	0.224	0.148^c^	0.703

*Note:* BMI: body mass index; FSH: follicle-stimulating hormone; LH: luteinizing hormone; T: testosterone; TSH: thyroid-stimulating hormone; AMH: anti-Mullerian hormone.

a N-PCOS vs N-CON.

b O-PCOS vs O-CON.

c N-PCOS vs O-PCOS.

d N-CON vs O-CON.

### Identification and Functional Analysis of Differentially Expressed Genes

**FIGURE 1:**
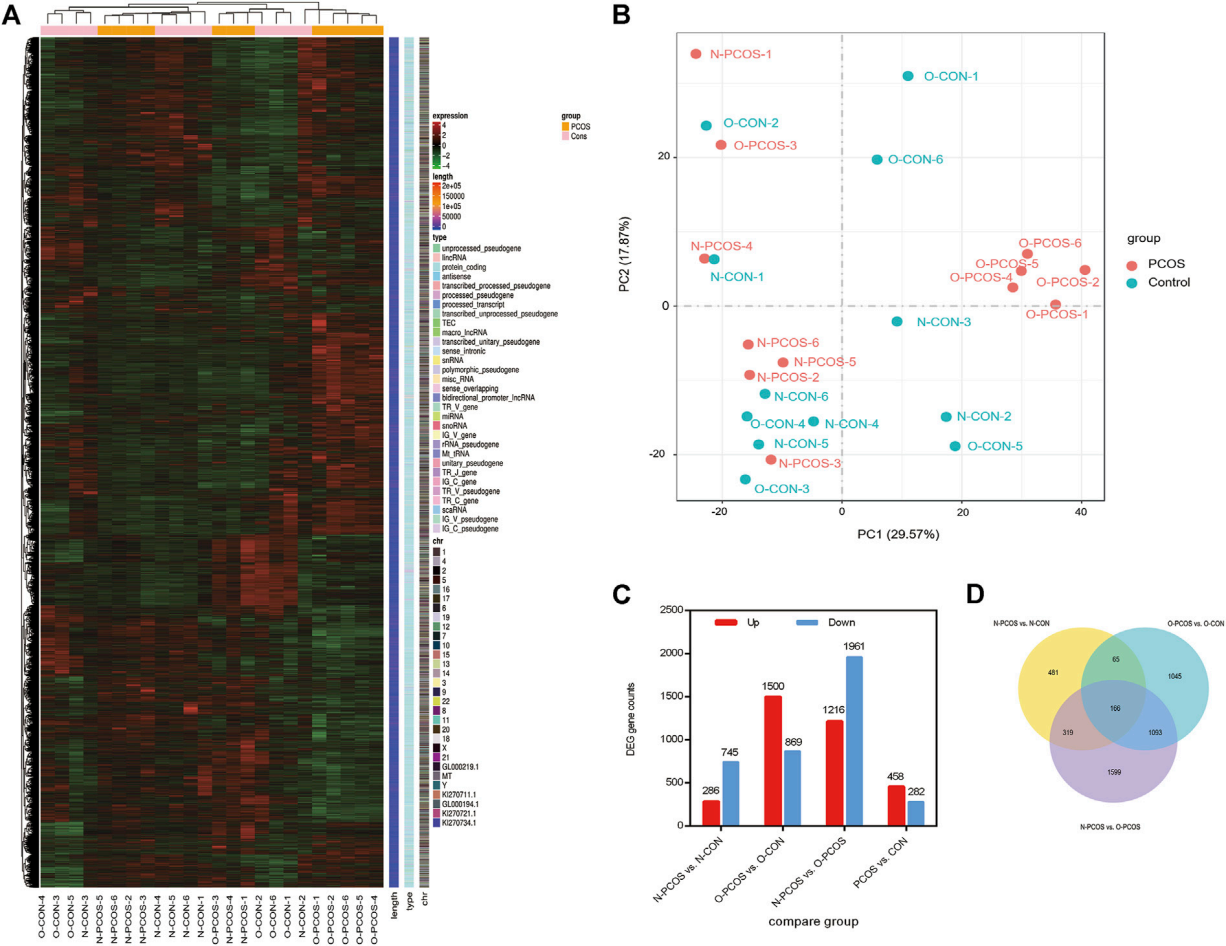
Changes in gene expression profiles of the endometrium between PCOS and controls. **(A)** Unsupervised hierarchical clustering of all gene expression in 24 subjects, six normoweight PCOS (N-PCOS), six overweight/obese PCOS (O-PCOS), six normoweight controls (N-CON), and six overweight/obese controls (O-CON). **(B)** PCA plot of RNA-Seq transcriptomes in the endometrium between PCOS and controls. **(C)** Numbers of DEGs in pairwise comparisons. **(D)** Venn diagram showing the number of DEGs in each comparison, and the overlaps between the three main comparison groups.

To better investigate the endometrial factors in the low fertility of patients with PCOS, we conducted a comparative transcriptomic analysis of the endometrium between 12 patients with PCOS (six normoweight and six overweight/obese) and 12 controls (six normoweight and six overweight/obese). Raw reads in the FASTQ format from replicated RNA-Seq libraries, three each for the PCOS patients and controls, were obtained, and their qualities were assessed using FastQC. There was an average of 45.84 and 45.18 M reads in PCOS patients and controls, respectively. After trimming and filtration, 97.68% of input reads from PCOS patients and 96.92% input reads from controls were retained as excellent quality sequences (Supplementary Table S2). First, we explored whether PCOS and control patients could be separated according to transcriptome profiling. After normalization and scaling of gene expressions, unsupervised hierarchical clustering was performed for all samples (Figure 1A). Most PCOS with normoweight and overweight/obese samples were clustered into two sub-trees, while control samples could not be grouped and were clustered within PCOS samples. Through principal component analysis (PCA), the clusters were different between overweight/obese and normoweight PCOS patients (Figure 1B). A total of DEGs in the endometrium were identified through pairwise comparisons, of which 1,031, 2,369, 3,177, and 740 DEGs were found between normoweight PCOS and control, overweight/obese PCOS and control, normoweight PCOS and overweight/obese PCOS, total PCOS and control, respectively (Figure 1C). The Venn diagram describes the intersection and union of DEGs in each comparison group (Figure 1D). The numbers in the overlapping parts of the circles represent the number of differentially expressed genes in each comparison.

**FIGURE 2:**
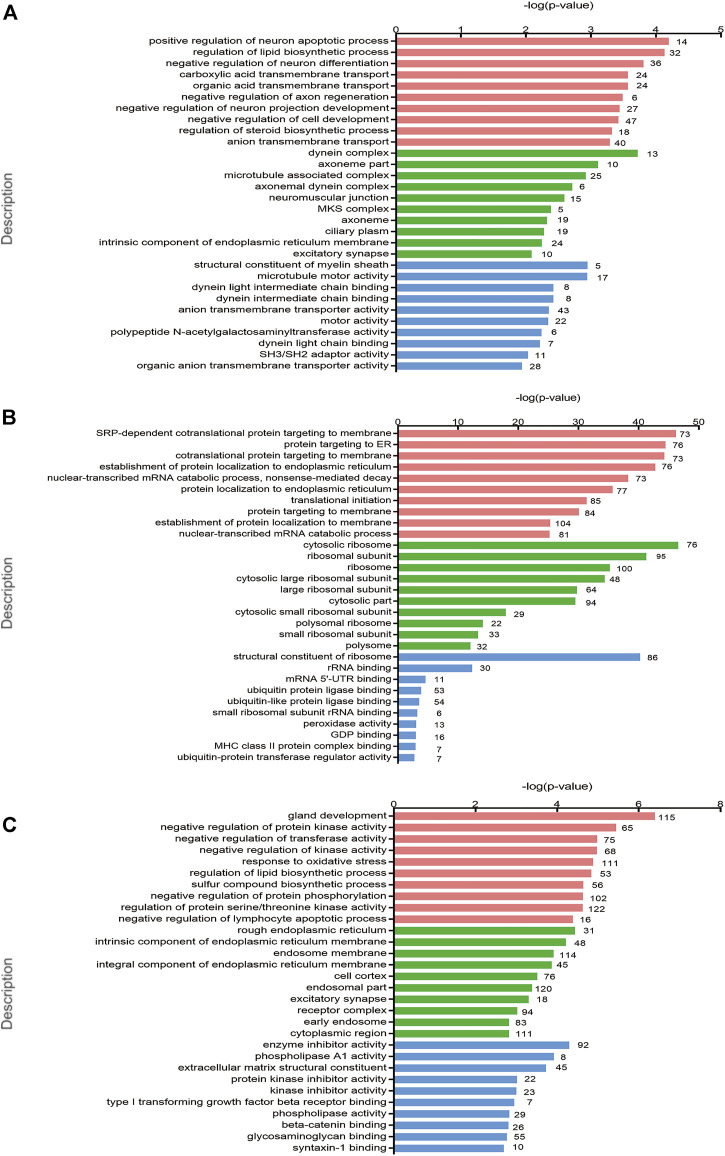
GO and KEGG pathway enrichment analyses of the differentially expressed genes. **(A–C)** Top 10 GO terms of gene enrichment analysis in PCOS versus control, normoweight PCOS versus normoweight control, and overweight/obese PCOS versus overweight/obese control in three main categories. The red columns indicate the biological process category, the green columns indicate the cellular component category, and the blue columns indicate the molecular function category.

GO enrichment analysis was applied for the identification of key genes and pathways, which contributed most to the different transcriptomic patterns between PCOS and controls. GO enrichment analyses of differentially biological processes, cellular components, and molecular functions were performed to evaluate the functional significance of these candidate genes. Positive regulation of neuron apoptotic process, regulation of lipid biosynthetic process, and negative regulation of neuron differentiation were the top three biological processes between women with PCOS and controls (Figure 2A). The localization of proteins encoded by genes showed high enrichment in cytosolic ribosomes indicated by cellular component terms between normoweight PCOS and normoweight controls (Figure 2B), whereas high enrichment in the rough endoplasmic reticulum (ER) between overweight/obese PCOS and overweight/obese controls (Figure 2C).

### Construction of Weighted Gene Co-Expression Networks and Identification of Key Modules

**FIGURE 3:**
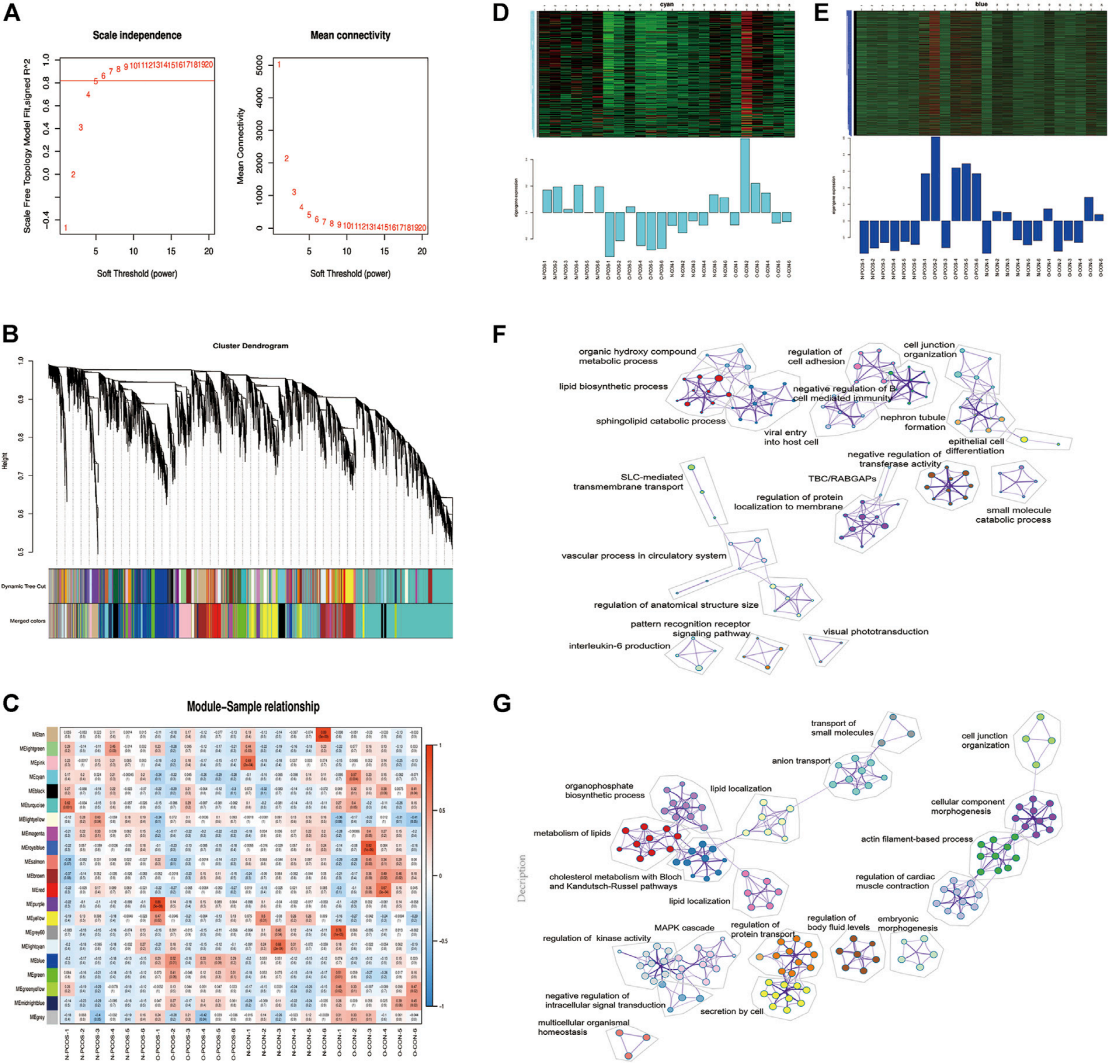
Weighted co-expression network construction and identification of key modules. **(A)** Determination of soft threshold power. **(B)** Dendrogram of all DEGs clustered based on the measurement of dissimilarity. The color band shows the results obtained from the automatic single block analysis. **(C)** Heatmap of the correlation between the module eigengenes and endometrium samples of PCOS and controls. **(D,E)** Heatmap of the modular dynamic gene expression patterns. **(F,G)** Network of enriched GO terms of genes containing in the cyan and blue modules, colored by GO cluster ID.

WGCNA was performed to construct co-expressed networks and identify co-expression modules. After selecting the determination of soft-thresholding power (*R* ^2^ = 0.8), hierarchical clustering of the samples was performed based on a Euclidean distance computed on log_10_-transformed RNA-Seq fractional counts. We profiled 17,593 genes from these 24 samples using WGCNA, and 20 gene co-expression modules were identified (Figures 3A,B). For each module, the gene co-expression was summarized by the eigengene, and we found that the cyan module was correlated with normoweight PCOS, while the blue module was significantly correlated with overweight/obese PCOS (Figure 3C). The modular dynamic gene expression pattern also showed that genes in the cyan module were upregulated in normoweight PCOS, whereas genes in the blue module were upregulated in overweight/obese PCOS (Figures 3D,E). To further clarify the biological functions of hub genes, GO functional enrichment of hub genes in cyan and blue modules was analyzed using Metascape (Zhou et al., 2019). Interestingly, the results showed that both hub genes in the cyan module, which related to normoweight PCOS, and hub genes in the blue module, which related to overweight/obese PCOS, enriched with lipid metabolism (Figures 3F,G). Furthermore, as shown in Figure 3F, the hub genes in the cyan module were also enriched in interleukin-6 production. Overall, these results suggested that abnormal metabolism, especially lipid metabolism, may have an essential impact on the endometrium of PCOS.

### Validation of Hub Gene Expression Associated With Endometrial Homeostasis

**FIGURE 4:**
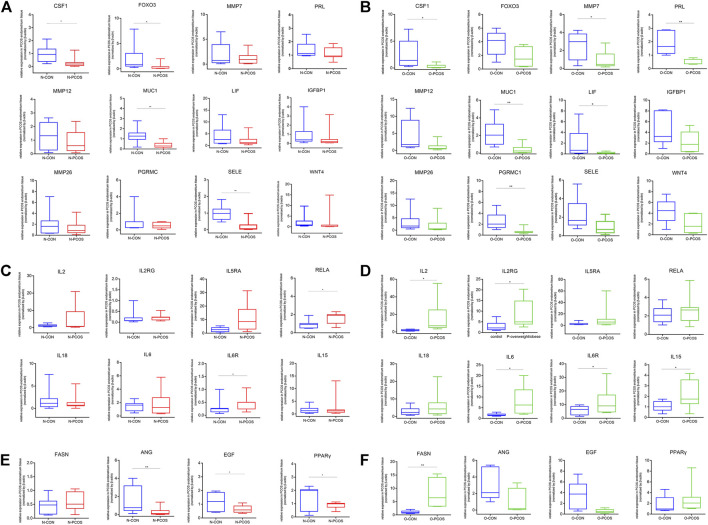
Validation of selected genes by quantitative RT-PCR. Quantitative RT-PCR was performed to compare the gene expression levels of 38 subjects, including 13 N-PCOS, 9 O-PCOS, 10 N-CON, and 6 O-CON. *Y*-axis indicates the relative expression scale. The data are presented in a boxplot. **(A)** Endometrial receptivity and decidualization-related gene expressions between N-PCOS and N-CON. **(B)** Endometrial receptivity and decidualization-related gene expressions between O-PCOS and O-CON. **(C)** Inflammatory gene expressions between N-PCOS and N-CON. **(D)** Inflammatory gene expressions between O-PCOS and O-CON. **(E)** Endometrial angiogenesis and energy metabolism-related gene expressions between N-PCOS and N-CON. **(F)** Endometrial angiogenesis and energy metabolism-related gene expressions between O-PCOS and O-CON. β-Actin was used as the internal control gene, and the expression level was evaluated by using the ΔΔCt method. ^*^ *p* < 0.05, ^**^ *p* < 0.01, and ^***^ *p* < 0.001.

Following the results of WGCNA, we selected 24 representative DEGs for real-time PCR validation on all 38 samples to comprehensively investigate the consequence of PCOS and obesity on endometrial homeostasis. The endometrium undergoes a rapid cycling process of proliferation, differentiation, and cell death under the influence of the ovarian hormonal milieu, and dysregulated gene clusters involved in metabolism, inflammatory response, and decidualization could alter endometrial homeostasis. These DEGs included those involved in decidualization (PRL, IGFBP1, and WNT4), endometrial receptivity (CSF1, LIF, MUC1, FOXO3, PGRMC1, MMP12, MMP7, MMP26, and SELE), angiogenesis (ANG and EGF), metabolism (FASN and PPARγ), and inflammatory response (IL2, IL6, IL18, IL6R, IL2RG, IL5RA, RELA, and IL15). In the qRT-PCR analysis, several endometrial receptivity-related and decidualization-related gene expressions were downregulated, whereas inflammatory gene expressions were upregulated in the PCOS group compared with control women regardless of the normoweight or overweight/obese group (Figures 4A–D). Furthermore, angiogenesis gene EGF and ANG expression were found significantly decreased in normoweight PCOS compared with BMI-matched controls. Energy metabolism-related genes also showed significant differences between PCOS and the control group (Figures 4E,F). However, it is worth noting that the qRT-PCR results of several genes might show variation because of the heterogeneity of specimens. The significance of this finding and its potential role in PCOS pathophysiology, especially endometrial factors, are currently under investigation.

### Functional Enrichment Analysis of DEGs Between Normoweight and Overweight/Obese PCOS

**FIGURE 5:**
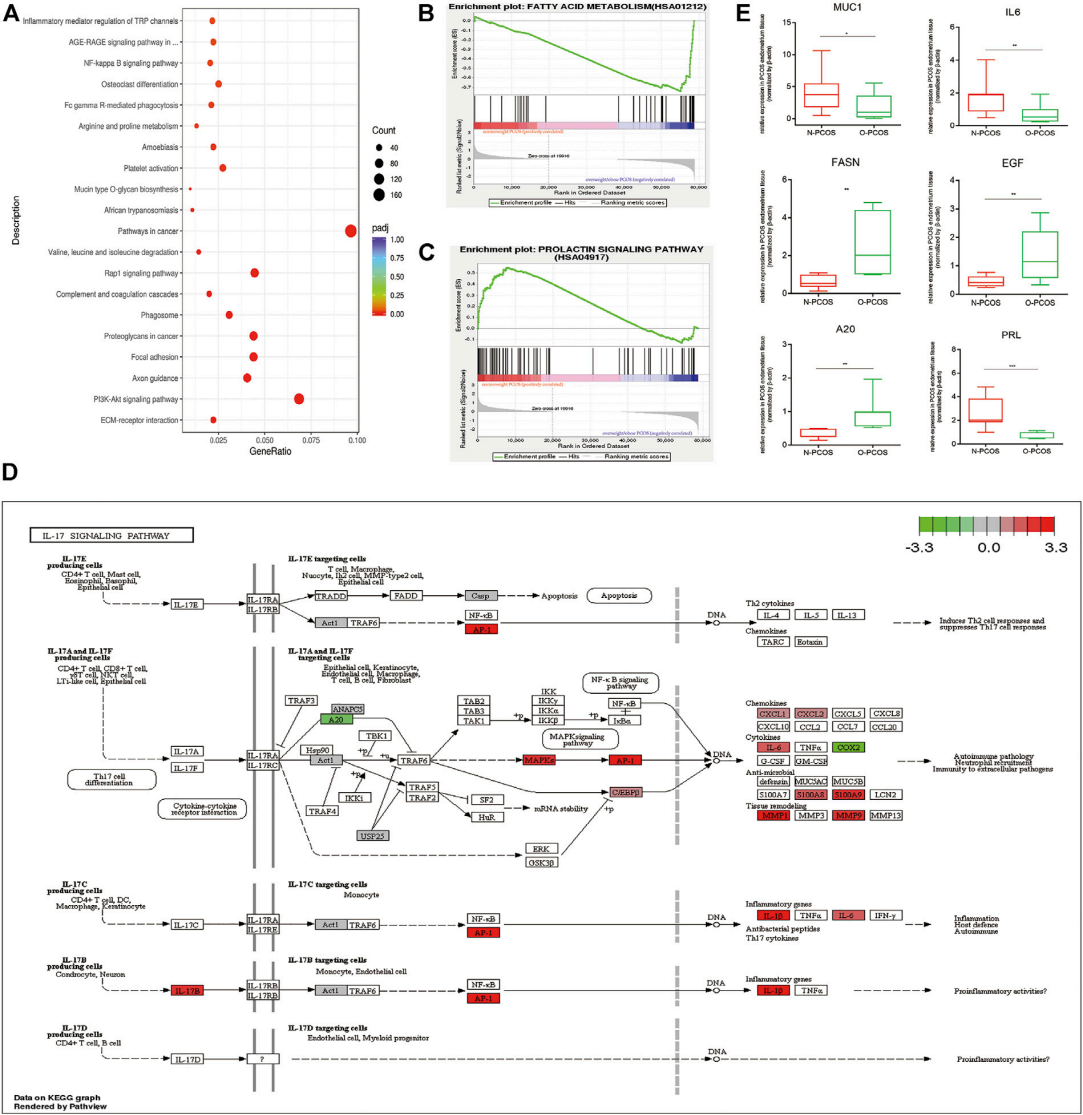
Functional enrichment analysis of the DEGs between normoweight and overweight/obese PCOS patients. **(A)** Statistics of KEGG pathway enrichment. The bubble size represents the count of genes annotated by the differential gene, and the color corresponds to the *p*-value. **(B,C)** GSEA enrichment score curves. **(D)** IL-17 signaling pathway constructed by KEGG. Red boxes represent upregulated genes in normoweight PCOS samples, and green boxes represent downregulated genes in normoweight PCOS samples. **(E)** Validation of the mRNA expression level by qRT-PCR.

The subfertility of women with PCOS may be heightened by the effect of obesity, metabolic, endocrine, and inflammatory abnormalities in ovulatory function, oocyte quality, and endometrial receptivity. The term endometrial receptivity was introduced to define the state of the endometrium during the window of implantation, and multiple markers of endometrial receptivity such as prolactin, interleukin, and mucin may help identify differences between fertile and subfertile populations. Aberrant hormonal contexts and metabolic phenotypes of patients with PCOS could alter endometrial homeostasis via dysregulated expression of gene clusters. The ECM–receptor interaction, PI3K-Akt signaling pathway, and axon guidance were the top three signaling pathways indicated by the KEGG analysis between normoweight PCOS and overweight/obese PCOS (Figure 5A). To explore the effect of obesity on endometrial phenotypes and functions during the time of implantation, a gene set enrichment analysis (GSEA) was performed. The data showed that the genes annotated as part of the fatty acid metabolism were remarkably enriched in the overweight/obese PCOS group compared with the normoweight PCOS group (Figure 5B), indicating there may be different metabolic patterns of the endometrium between the two groups. Moreover, the expression levels of the prolactin signaling pathway were significantly enriched in the normoweight PCOS compared with the overweight/obese PCOS group, suggesting a significant difference in endometrial decidualization between the two groups (Figure 5C). The comparison of normoweight versus overweight/obese PCOS yielded a group of genes related to the IL-17 signaling pathway whose transcript levels were upregulated in the normoweight PCOS group. KEGG pathway analysis showed that several central components of the IL-17 signaling pathway were highly expressed in normoweight PCOS compared to overweight/obese PCOS (Figure 5D). Additionally, the qRT-PCR results were in line with our RNA sequencing analysis, showing that MUC1, IL6, and PRL were significantly downregulated, whereas FASN, EGF, and A20 were upregulated in overweight/obese PCOS compared with normoweight PCOS patients (Figure 5E). Therefore, our study highlighted the differences between normoweight and overweight/obese PCOS, demonstrating that obesity might influence the endometrial receptivity through dysregulated metabolic pattern, decidualization, and inflammatory state.

## Discussion

This is the first report of differential gene expression profiles in endometrium taken from different types of PCOS and non-PCOS patients based on their BMI. PCOS is a complex endocrine disease with elevated systemic levels of LH, androgens, insulin resistance, and cytokines suggestive of a pro-inflammatory state. However, the roles of these components at the endometrium level remain understudied, partly due to the limited tissue availability and the diverse PCOS patient phenotypes. In this study, we show that 1) endometrium from PCOS patients express significantly different transcripts encoding endometrial receptivity, inflammatory response, angiogenesis, and energy metabolism; 2) normoweight PCOS and overweight/obese PCOS patients define two distinct PCOS disease subtypes.

BMI is recommended as a surrogate marker of excess adiposity in terms of overweight and obesity (Nagaya et al., 1999; Frankenfield et al., 2001). Although an ideal alternative is to use actual measures of fatness, rather than body mass, previous studies have shown that the correlations between the BMI and body fat percentage were generally good in Chinese people (Wang et al., 2010). Moreover, Chinese people tend to have lower BMI but higher fat volume and obesity-related metabolic risks are greater in Asian people than in European populations (WHO Expert Consultation, 2004). Thus, we divided the patients into normoweight group and an overweight/obese group according to Asia-specific BMI criteria, which were determined by the World Health Organization Western Pacific Region (WHO Expert Consultation, 2004).

The following discussion relates to the list of DEGs with a |FC|≥ 2, which provides a detailed comparison of the groups. The comparisons showed that the largest number of DEGs were the normoweight PCOS versus overweight/obese controls (3,177) and the overweight/obese PCOS versus overweight/obese control (2,369). The comparison of normoweight PCOS with normoweight controls resulted in a smaller number of DEGs (1,031). Comparison of the entire groups of PCOS patients with the entire groups of controls, irrespective of weight, yielded the smallest group of DEGs (740). These findings suggest that the most unique groups in these comparisons were the overweight/obese PCOS. The expression of the genes in this group’s endometrium was very different from both the normoweight PCOS and the same-weight controls. The smaller number of differential genes in the more general comparison of all PCOS with all controls suggests that the difference between PCOS and non-PCOS endometria becomes less pronounced when weight is disregarded. Taken together, our results may suggest that normoweight PCOS and overweight/obese PCOS patients define two distinct PCOS disease subtypes.

The PCA data showed a non-significant cluster between the different subgroups. This may be due to the small sample size in this study and the heterogeneity of PCOS women. Additionally, lack of body fat measurement may also contribute to the heterogeneity observed in PCA as BMI cannot distinguish between body fat and muscle mass. Previous studies also demonstrated that normoweight PCOS women with central obesity had higher risks of insulin resistance and dyslipidemia than those without central obesity, indicating the influence of BMI and central obesity simultaneously on metabolic dysfunctions of PCOS (Mu et al., 2018).

Hypothalamus–pituitary–ovary axis imbalance is considered important in the pathophysiology of PCOS, indicating that central modulation, especially the abnormal activation of GnRH neurons, contributes to PCOS development (Liao et al., 2021). Increased GnRH pulse frequency and LH pulse frequency are associated with ovarian dysfunction and reproductive disorders (Tsutsumi and Webster, 2009). Meanwhile, impaired steroid hormone feedback to GnRH neurons drives hyperactivity of the neuroendocrine axis, forming a vicious cycle in PCOS (Ruddenklau and Campbell, 2019). Our sequencing data showed that the biological processes including positive regulation of the neuron apoptotic process and negative regulation of neuron differentiation were differentially expressed between PCOS and controls. However, whether these genes involved in the regulation of neurons could affect the endometrial function of PCOS remains to be investigated by further research.

The endoplasmic reticulum contributes to the synthesis, folding, modification, and trafficking of secretory and cell surface proteins, which generally contain large numbers of attached ribosomes (granular or rough ER). Many studies have shown that ER homeostasis plays an important role in the maintenance of normal pregnancy, considering that the reproductive tissues undergo highly dynamic cellular, molecular, and genetic changes during this process (Guzel et al., 2017). Conversely, the contribution of impaired ER homeostasis to the pathogenesis of various reproductive diseases including endometriosis, recurrent pregnancy loss, and preterm birth has also been reported. Consistently, our data showed that ER- and ribosome-related genes were differentially expressed between PCOS and controls, indicating that ER and ribosome signaling mechanisms are involved in the development of PCOS. However, further studies are required to investigate the underlying mechanisms.

A previous study showed that endometrial cells from PCOS patients, particularly endothelial and mesenchymal stem cells, revealed differences in inflammatory and cancer-related genes (Piltonen et al., 2013). Using an integrative transcriptomic data analysis, Kori et al. (2016) found that PCOS, endometriosis, and ovarian cancer shared common signatures. Our study identified that pathways in cancer were enriched terms in KEGG analysis and highlighted the critical role of these genes in PCOS. The top 10 significant GO and KEGG pathways belonged to neuron activity, transmembrane transport, gland development, and steroid biosynthesis, which is reflective of the cellular changes and biological activity occurring in the endometrium. However, the objective of this study was to determine global changes in gene expression related to endometrial receptivity, inflammatory response, angiogenesis, and energy metabolism. The WGCNA corresponds to a data reduction and unsupervised classification method, which allowed for identifying the pattern of correlation among genes (Langfelder and Horvath, 2008). According to the WGCNA algorithm, we found strong correlations of module blue with overweight/obese PCOS. These results demonstrated that key genes within the blue module may serve as potential markers of obesity or PCOS. We determined that the genes in the blue module were enriched in lipid metabolism, which is closely related to the endometrial function.

The term “endometrial receptivity” refers to the ability of the endometrium to undergo changes that will allow the blastocyst to attach, penetrate, and induce localized changes in the stroma (Rashid et al., 2011). Identifying the specific endometrial molecular milieu of PCOS patients may provide clues to better understand the etiology of PCOS. In our study, leukemia inhibitory factor (LIF), a critical factor for implantation, is downregulated in both normoweight and overweight/obese PCOS patients compared with same-BMI controls, confirmed by qRT-PCR. Previous reports have shown that low expression of LIF was associated with poor IVF outcomes (Serafini et al., 2009). Consistent with our results, LIF expression was found to be downregulated in the PCOS mouse uterus (Li et al., 2016). Thus, we hypothesize that the abnormal expression of endometrial receptivity-related genes may contribute to the high rates of pregnancy complications in PCOS. Additionally, our results showed that mucin-1 (MUC-1) and colony-stimulating factor-1 (CSF-1) were downregulated in PCOS patients. Consistently, altered expression of MUC-1 was found in hyperandrogenic women with PCOS (Margarit et al., 2010). Previous studies showed that fertile women exhibited a higher level of endometrium MUC-1 expression than infertile patients (Margarit et al., 2010). CSF-1 was downregulated in the endometrium of patients with recurrent miscarriages, and increased serum CSF-1 levels were associated with increased pregnancy rates (Othman et al., 2012). Therefore, the reduced expression of multiple receptivity-associated genes, LIF and MUC-1, in the PCOS endometrium may account for the observed reduction in cycle fecundability in these women.

During the progesterone-dominated “implantation window,” endometrial stromal cells undergo morphological and functional changes to differentiate into decidual cells, which are receptive to embryonic implantation. Abnormalities in the decidualization process may be the pathophysiologic basis of several pregnancy complications. In our study, prolactin (PRL), which is markedly induced during human uterine decidualization, was downregulated in overweight/obese PCOS. GSEA also showed that the expression level of the prolactin signaling pathway was significantly enriched in the normoweight PCOS group compared with the overweight/obese PCOS group, suggesting that adiposity might affect endometrial decidualization in PCOS.

The EGF pathways are important for both implantation and differentiation of the uterus (Ejskjaer et al., 2005). Endometrial angiogenesis is believed to be regulated by angiogenic growth factors, including vascular endothelial growth factor (VEGF), epidermal growth factor (EGF), and fibroblast growth factor 2 (FGF-2), as well as angiogenin (ANG). Previous studies have indicated that multiple angiogenic factors are dysregulated in PCOS (Tal et al., 2015). Mouse studies indicate that the hyperandrogenic environment of PCOS may lead to decreased angiogenesis in the uterus (Gopalakrishnan et al., 2016; Gong et al., 2019). Human studies also demonstrate that uterine artery resistance is increased, and the endometrial blood flow indices are impaired in hyperandrogenic PCOS women (Chekir et al., 2005; Lam et al., 2009). Consistently, EGF and ANG were found to be significantly decreased in normoweight PCOS patients in our study. We hypothesize that decreased angiogenesis may contribute to placental abnormalities of PCOS, which may further lead to an increased incidence of pregnancy complications.

Our RNA sequencing analyses demonstrate that both normoweight and overweight/obese PCOS patients present an increased endometrial immune/cytokine response. Several central components of the IL-17 signaling pathway exhibited higher expression in normoweight PCOS women than in overweight/obese PCOS, suggesting that the PCOS condition itself defines a propensity for a pro-inflammatory endometrial milieu. The data on the endometrial immune environment in women with PCOS are limited. However, it is well known that PCOS is associated with systemic and local low-grade inflammation (Escobar-Morreale et al., 2011; Hu et al., 2020). Our previous study has reported the PCOS endometrium presenting with an altered immune cell profile (Liu et al., 2021). Moreover, several isolated endometrial cell populations in women with PCOS showed upregulation of inflammatory genes, indicating an altered inflammatory potential of endometrial cells in these patients (Piltonen et al., 2013). Consistent with our qRT-PCR, results showed that IL-2, IL-2R, IL-6, IL-6R, and IL-15 expressions were significantly higher in overweight/obese PCOS than in BMI-matched controls. These studies together reveal an endometrial pro-inflammatory phenotype in women with PCOS. Additionally, increased inflammation also induces effects on molecules of the insulin signaling pathway in the endometrium of PCOS women, which could partly explain the adverse pregnancy outcomes in these patients (Orostica et al., 2016).

Energy metabolism is crucial for proper endometrial function as the vast turnover of endometrial tissue growth, differentiation, and renewal. Lipid abnormalities play an important role in the development of PCOS. Fatty acid synthase (FASN), the key enzyme of fatty acids synthesis, was found to increase in adipose tissues of PCOS rats (Lang et al., 2019). Consistently, our results showed that FASN expression was significantly higher in the overweight/obese PCOS endometrium. GSEA also showed fatty acid metabolism was remarkably enriched in the overweight/obese PCOS group compared with the normoweight PCOS group. Moreover, free fatty acid accumulation was detrimental to the endometrium, and palmitic acid was shown to impair endometrial decidualization (Rhee et al., 2016). Thus, our results suggest that metabolic disruption of the endometrium may perturb normal decidualization of PCOS.

Insulin resistance is a common metabolic feature of PCOS. It has been well documented that women with PCOS, independent of obesity, are insulin-resistant and have compensatory hyperinsulinemia (Dunaif et al., 1989). Thus, we examined the expression of the molecules involved in the insulin pathway in the endometrium of women with PCOS and found that peroxisome proliferator-activated receptor-γ (PPARγ) was decreased in women with normoweight PCOS compared with controls. PPARγ, a nuclear transcription factor, plays a pivotal role in glucose and lipid metabolism and influences insulin sensitivity (Feng et al., 2017; Fernandez et al., 2017). Previous studies also showed decreased expression of PPARγ mRNA in ovarian granulosa cells in PCOS, which may be involved in the pathogenesis of this disease (Cao et al., 2019). Our work further suggests that insulin resistance in the endometrium and derangement in glucose and lipid metabolism likely contribute to defects in endometrial receptivity in PCOS.

Our results suggest that PCOS patients who do not suffer from the comorbidity of obesity represent the authentic syndrome with its unique characteristics. Obesity may be considered as a modifier of the syndrome or as a separate pathological mechanism that results in similar consequences. The fact that a comparison of the entire PCOS group with healthy controls revealed significantly fewer DEGs implies that PCOS in normoweight and overweight/obese individuals should be regarded as separate subentities. Further large-scale molecular studies and subgrouping PCOS patients into more homogeneous groups may provide a better understanding of the molecular pathophysiology of this syndrome.

The strength of this study includes the comparison of the endometrium at a specific moment (mid-secretory phase) in different subgroups of PCOS women and controls. First, the limitations of the study include different methods of collecting mid-secretory phase endometrial tissue samples, which were hormone replacement therapy cycles in PCOS women who were anovulatory, and natural cycles in controls. This method cannot avoid the influence of different treatments on the endometrium, although H&E staining was used to confirm the histologic phase. Second, this study cannot provide direct support for the role of an “endometrial factor” as a contributor to the pathogenesis of infertility of PCOS as conception was avoided due to the endometrial biopsy; the performance of an *in vitro* implantation model may be of interest in the future to adequately interpret the relationship between endometrial function and the physiological process of pregnancy. Third, the sample size was small, which may bias the interpretation of the results, especially when the heterogeneities in phenotypes, comorbidities, and severities in PCOS were taken into account. Last, more controls could have been established, such as normoweight and overweight/obese controls. However, the difficult recruitment of these patients and the complicated interpretation of the results make these comparisons possible targets for further study.

In summary, we present a list of significant differences in gene expression and have validated important examples in the PCOS endometrium that demonstrate LIF, MUC-1, and CSF-1 reduced endometrial receptivity, EGF and ANG decreased angiogenesis, IL-2, IL-2R, IL-6, IL-6R, and IL-15 increased inflammation, FASN perturbed energy metabolism, and PPARγ increased insulin resistance. Herein, our data support that the pathogenesis of PCOS may relate to an “endometrial factor.” The observed differences in gene expression provide a roadmap for future studies to examine the efficacies of various targeted therapies for PCOS that could improve endometrial function, improve fertility, and reduce the risk for pregnancy complications.

## Data Availability

All data generated or analyzed during this study are included in this published article or in the data repositories listed in References. Gene expression data were submitted to the NCBI Database (BioProject ID: PRJNA777962).
