# Supplementary material for: Transcriptomic Analysis Reveals Endometrial Dynamics in Normoweight and Overweight/Obese Polycystic Ovary Syndrome Women
Source: Front Genet. 2022 May 13;13:874487. doi: 10.3389/fgene.2022.874487 (PMC9136323; doi:10.3389/fgene.2022.874487)
Supplement: Supplementary file 1 [file Table2.DOCX]

Table S1 Related primer sequences.

| Genes | Primer sequences (5′–3′) | Reverse primer (5′–3′) |
| --- | --- | --- |
| EGF | TTTATGAAAGCCTCAGATGGGA | CTTTAGATCAACTTCACCACCTG |
| FASN | CAACCTCTCCCAGGTATGC | TGCTGATGATGGACTCCAG |
| IL2RG | GAGTACATGAATTGCACTTGG | CGAGTTCTTGTACCAATAATGC |
| IL6R | CATAGTGTCCATGTGCGTC | ATCAGGCTGCAAGATTCCA |
| PGRMC1 | GACCTTTCTGACCTCACTG | CACGTGATGATACTTGAAAGTG |
| PPARG | TTCCATTCACAAGAACAGATCC | CTTTGATTGCACTTTGGTACTC |
| PRL | CAAAGCTGTAGAGATTGAGGAG | GTTTCAGGATGAACCTGGC |
| RELA | GCTTGTAGGAAAGGACTGC | AGGTTCTGGAAACTGTGGA |
| SELE | CGTGGAGATCTACATCAAGAG | GTATTGGTACAGGCAGCTG |
| MMP7 | TAGTTGGGGGACTGCGGATA | CAGGAAGTTCACTCCTGCGT |
| MMP26 | GGATGATGACGCCACTCACA | GGTACACAACTTGGGCCACT |
| MMP12 | TGCAGCAGTTCTTTGGGCTA | AGATGCTGTACATCGGGCAC |
| CSF1 | CTCTCCGCATCCCAGGACAG | AGACCAACAACAGCAGGGAG |
| MUC1 | CACAGGACCCAAGAAGTCCC | CGTGGAGGAGGTCTGCATTT |
| IL15 | CTCTGCGCCCAAAAGACTTG | TGCTTTGAAGAGCCAGAGGG |
| IL18 | GAAACGTCAATAGCCAGTTGC | TCCCATGCTCTTTCTCACAACA |
| IL5RA | CAAATGGGCGGTAGGCGTG | CCAGGTTTCCGGGCCCTCAC |
| WNT4 | AGGAGGAGACGTGCGAGAAA | CGAGTCCATGACTTCCAGGT |
| IL6 | AGACAGCCACTCACCTCTTCAG | TTCTGCCAGTGCCTCTTTGCTG |
| IL2 | AGAACTCAAACCTCTGGAGGAAG | GCTGTCTCATCAGCATATTCACAC |
| ANG | ATTCAGCGACGTGAGGATGGCA | GCACATAGCGTTGCTGATTAGTC |
| FOXO3 | GGACCCGCATGAATCGACTAT | CGGACAAACGGCTCACTCT |
| IGFBP1 | TTGGGACGCCATCAGTACCTA | TTGGCTAAACTCTCTACGACTCT |
| LIF | GCTATGTGCGCCTAACATGA | AGTGGGGTTCAGGACCTTCT |
| A20 | CATTGTTCTCGGCTATGACAG | GGAACAGCTCGGATTTCAG |
| ACTB | CCTTGCACATGCCGGAG | GCACAGAGCCTCGCCTT |

Table S2 RNA-sequencing QC data.

| sample | raw reads | clean reads | clean bases | error rate |
| --- | --- | --- | --- | --- |
| N-PCOS-1 | 45787942 | 44715714 | 6.71G | 0.03 |
| N-PCOS-2 | 46216314 | 45521568 | 6.83G | 0.03 |
| N-PCOS-3 | 45440044 | 44405212 | 6.66G | 0.03 |
| N-PCOS-4 | 45316246 | 44314216 | 6.65G | 0.03 |
| N-PCOS-5 | 46085962 | 44928990 | 6.74G | 0.03 |
| N-PCOS-6 | 46467274 | 45585016 | 6.84G | 0.03 |
| O-PCOS-1 | 45899138 | 44864370 | 6.73G | 0.03 |
| O-PCOS-2 | 47564930 | 46558414 | 6.98G | 0.03 |
| O-PCOS-3 | 45576582 | 44646994 | 6.7G | 0.03 |
| O-PCOS-4 | 45776618 | 44850448 | 6.73G | 0.03 |
| O-PCOS-5 | 45727112 | 44691182 | 6.7G | 0.03 |
| O-PCOS-6 | 44303472 | 42315888 | 6.35G | 0.03 |
| N-CON-1 | 46055080 | 45318494 | 6.8G | 0.03 |
| N-CON-2 | 45469386 | 44923890 | 6.74G | 0.03 |
| N-CON-3 | 42923108 | 42331530 | 6.35G | 0.03 |
| N-CON-4 | 45384744 | 44584852 | 6.69G | 0.03 |
| N-CON-5 | 47405098 | 46651652 | 7.0G | 0.03 |
| N-CON-6 | 47456292 | 46754958 | 7.01G | 0.03 |
| O-CON-1 | 42474132 | 40506906 | 6.08G | 0.02 |
| O-CON-2 | 45379974 | 43382984 | 6.51G | 0.02 |
| O-CON-3 | 44867586 | 42760598 | 6.41G | 0.03 |
| O-CON-4 | 44155668 | 42054062 | 6.31G | 0.03 |
| O-CON-5 | 45998236 | 43958370 | 6.59G | 0.02 |
| O-CON-6 | 44601312 | 42238772 | 6.34G | 0.02 |
